# Expanding the taxonomic range in the fecal metagenome

**DOI:** 10.1186/s12859-021-04212-6

**Published:** 2021-06-09

**Authors:** Theo R. Allnutt, Alexandra J. Roth-Schulze, Leonard C. Harrison

**Affiliations:** 1 Walter and Eliza Hall Institute of Medical Research, 1G Royal Parade, Parkville, Melbourne, VIC 3052 Australia; 2 Department of Medical Biology, University of Melbourne, Melbourne, VIC 3010 Australia

**Keywords:** Metagenomics, Eukaryotes, Viruses, Classifier, Benchmarking

## Abstract

**Background:**

Except for bacteria, the taxonomic diversity of the human fecal metagenome has not been widely studied, despite the potential importance of viruses and eukaryotes. Widely used bioinformatic tools contain limited numbers of non-bacterial species in their databases compared to available genomic sequences and their methodologies do not favour classification of rare sequences which may represent only a small fraction of their parent genome. In seeking to optimise identification of non-bacterial species, we evaluated five widely-used metagenome classifier programs (BURST, Kraken2, Centrifuge, MetaPhlAn2 and CCMetagen) for their ability to correctly assign and count simulations of bacterial, viral and eukaryotic DNA sequence reads, including the effect of taxonomic order of analysis of bacteria, viruses and eukaryotes and the effect of sequencing depth.

**Results:**

We found that the precision of metagenome classifiers varied significantly between programs and between taxonomic groups. When classifying viruses and eukaryotes, ordering the analysis such that bacteria were classified first significantly improved classification precision. Increasing sequencing depth decreased classification precision and did not improve recall of rare species.

**Conclusions:**

Choice of metagenome classifier program can have a marked effect on results with respect to precision of species assignment in different taxonomic groups. The order of taxonomic classification can markedly improve precision. Increasing sequencing depth can decrease classification precision and yields diminishing returns in probability of species detection.

**Supplementary Information:**

The online version contains supplementary material available at 10.1186/s12859-021-04212-6.

## Background

Metagenomics studies the genome content of populations of microorganisms. Advances in high throughput parallel sequencing now allow researchers to simultaneously sequence thousands of genomes. Methods to analyse such large and complex datasets are being continuously developed, attempting to keep pace with ever-growing data. Currently, there are two general approaches to metagenome analysis [1]. First, classification, which aims to identify sequence reads and assign them to a known species or taxa. This is achieved by alignment or mapping of reads or read fragments (k-mers) to databases of reference genes or genomes along with algorithms to select the best matches and decide the appropriate taxonomic level of assignment. Classified reads can then be counted in order to build up an abundance profile of the entire population under study. Second, assembly, which may also be used to classify and quantify genomes, but primarily aims to obtain discrete and complete genomes. These two approaches are usually used together to maximise information from metagenome data sets.

The gut microbiome is an important determinant of human health and is altered in response to diet and other environmental conditions, life events and disease states [2, 3]. However, almost all knowledge of the gut microbiome relates to its bacterial component, which comprises the majority of its genomic mass [4], although emerging evidence suggests that viruses, including bacteriophages [5], and fungi [6] also contribute to the functions of the gut microbiome. In addition to whole metagenome sequencing, methods that specifically target and enrich the virome have been developed [7]. The fungal component of the gut microbiome (the mycobiome) has been more widely studied using rRNA ITS amplicon sequencing [8], due largely to the computational problems presented by large eukaryotic genomes which tend to contain larger proportions of repetitive sequence compared to bacteria and viruses and may contain viral and bacterial sequences, by integration or contamination.

Metagenomic sequencing covers all DNA and therefore includes a small proportion of viral and eukaryotic (fungi, parasites and undigested food DNA) sequences that are often discarded or overlooked in analyses. The majority of viral sequence is expected to be retrovirus RNA (both host and bacteriophage), together with DNA viruses. The representation of the RNA virome can be improved with reverse-transcription prior to DNA metagenomic sequencing. Viral genomes are small and therefore can be represented in existing sequence classification databases. The large size of eukaryote genomes, however, means they are usually excluded from classifier databases. Recently, two classifier programs have been developed specifically for eukaryote classification in metagenomes. EukRep [9] requires genome assembly for eukaryote classification and is not applicable to fecal metagenomes because we expect the abundance of eukaryotic reads to be too low to allow assembly. CCMetagen [10] uses read-mapping to purportedly improve classification of eukaryotes and therefore may be applicable to low-abundance genomes. However, given optimised bacterial, viral and eukaryote databases it should also be possible to apply previously developed and more widely used metagenome classifiers to the total taxonomic content of metagenomes.

In this study, we aimed to identify the optimal program(s) and approaches for classifying the wider taxonomy of the fecal metagenome, but our findings could be extended to any mixed populations of bacteria, viruses and eukaryotes. We examined the performance of five metagenome classifiers for their ability to correctly classify metagenomes with abundance distributions of organisms expected in human fecal samples. Classifiers were chosen for differences in methodology, and all were expected to have adequate speed for processing large numbers of samples and acceptable memory requirements. We also examined the effect of order of analysis between bacteria, viruses and eukaryotes, with the aim of improving classification by excluding confounding sequences. Finally, the effect of sequencing depth on classification precision and probability of a species' detection was examined.

## Results

### Practical application considerations

The time taken for the programs being compared to classify 10 million 150 bp simulated reads is shown in Table 1. Classification was carried out in three separate steps for bacteria, viruses and eukaryotes. Speed was, as expected, proportional to database size; viruses were classified faster than bacteria and eukaryotes, except in the case of Centrifuge which took 44 s longer to classify viruses than bacteria. In total time taken, Centrifuge was the fastest classifier (19:29) followed by Kraken2 (20:24), BURST (28:20), CCMetagen (55:44) and UBLAST (30:23:09). Although not directly comparable to the other metagenome classifiers, MetaPhlAn2 (5:12) was the second fastest for bacteria. Classifiers differed in their speed by taxonomic group, Centrifuge being the fastest to classify bacteria and Kraken2 the fastest to classify viruses and eukaryotes.

**Table 1:** Time required by each program to classify 10 million 150 bp reads into each taxonomic group (hours:minutes:seconds)

Program	Bacteria	Viruses	Eukaryotes	Total
BURST	0:08:54	0:02:28	0:16:58	0:28:20
Kraken2	0:09:56	0:00:24	0:10:04	0:20:24
Centrifuge	0:02:01	0:02:45	0:14:43	0:19:29
Ublast	10:33:50	1:47:55	18:01:24	30:23:09
CCMetagen	0:04:47	0:00:28	0:50:29	0:55:44
MetaPhlAn2	0:05:12	–	–	–

24 CPUs (Intel(R) Xeon(R) Gold 6130 CPU @ 2.10 GHz)

Database size, shown in Table 2, varied up to 86-fold between classifiers. Virus databases were small and inconsequential with respect to modern RAM availability. Bacterial databases and especially eukaryotic databases would usually exceed available RAM for desktop computers. Centrifuge consistently built the smallest databases: 6.3, 0.057 and 115 GB for bacteria, viruses and eukaryotes, respectively. BURST required the largest bacterial database (48.2 GB) and Kraken2 required the largest eukaryotic database (218 GB). Eukaryotic databases were more consistent in size between programs than those for bacteria or viruses (less than two-fold difference in size between smallest and largest compared to > seven-fold difference for bacterial databases).

**Table 2:** Database size (GB) required for each program and taxonomic group

Program	Bacteria	Viruses	Eukaryotes
BURST	48.2	4.9	119
Kraken2	22	0.17	218
Centrifuge	6.3	0.057	115
Ublast	15	0.1	200
CCMetagen	17.3	0.19	163
MetaPhlAn2	0.65	–	–

In addition to these considerations, different complexities of scripting steps were required for each program. In particular, when processing sequence files by taxonomic group order, BURST and CCMetagen presented the most difficulties, requiring sequences not classified at each step to be programmatically extracted from the initial sequence file in order to be processed at the next step. This added time that is not included in Table 1. Kraken2 and Centrifuge provided the built-in option to output unclassified reads from each step which could then be used for the next step, greatly simplifying their pipelines. BURST also required the use of a separate, customised script to extract the LCA from the alignment output, unlike other programs which included this function within their main classifier code.

### Comparison of classifiers using an unordered approach

**Fig. 1:**
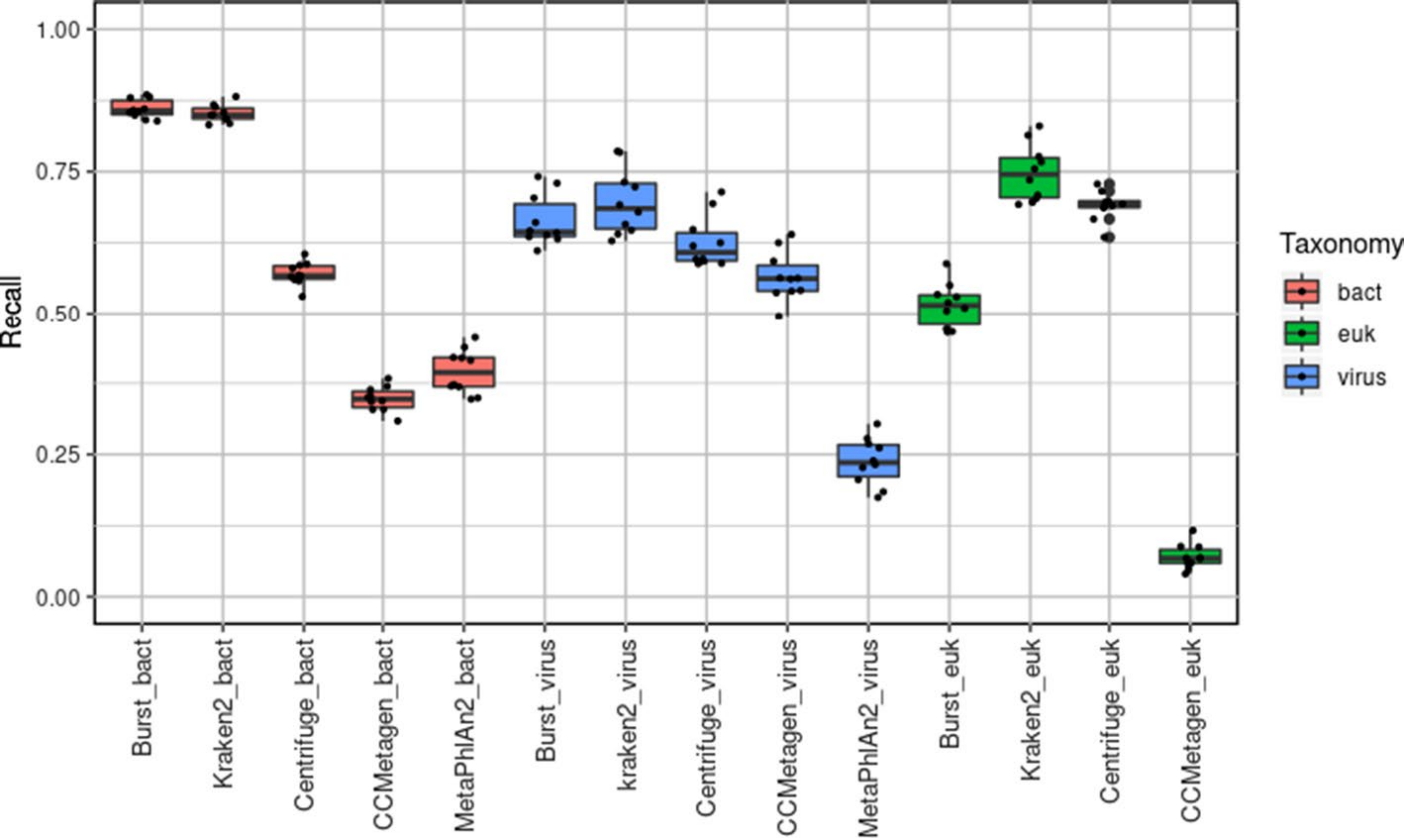
Recall determined by classifier programs using an unordered analysis approach

**Fig. 2:**
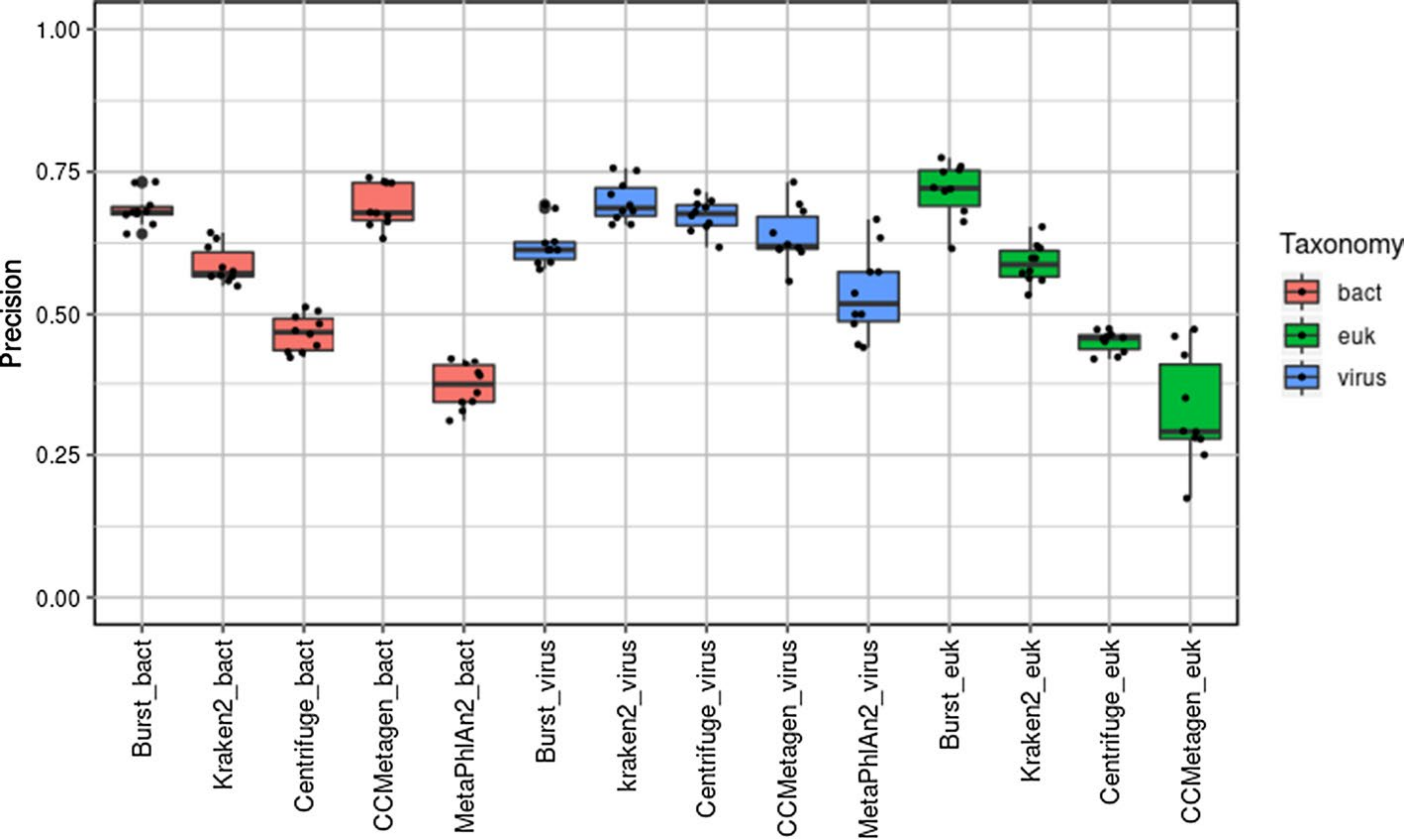
Precision determined by classifier programs using an unordered analysis approach

Recall and precision measurements for each program are shown in Figs. 1 and 2, respectively, using the unordered approach (bacteria, viruses and eukaryotes classified with the full set of reads for each). Unordered results are also summarised in Tables 3 and 4. Both measures showed large variation between programs and between taxonomic groups. Although not the primary focus of this study, bacterial classification was included for the purpose of comparison. The highest mean recall for bacteria was achieved similarly by BURST (0.860) and Kraken2 (0.852), followed by Centrifuge (0.571), MetaPhlAn2 (0.396) and CCMetagen (0.347), with *P*-values = 0.26, < 2 × 10^−16^, 2 × 10^−10^ and 0.003, respectively. For virus recall, BURST (0.664), Kraken2 (0.697) and Centrifuge (0.626) performed similarly and better than CCMetagen (0.566) or Metaphlan (0.237), with *P*-values = 0.171, 0.007, 0.007 and 1 × 10^−12^, respectively. For eukaryote recall, Kraken2 performed best (0.748), followed by Centrifuge (0.69), BURST (0.514) and CCMetagen (0.007), with *P*-values = 7 × 10^−10^, 6 × 10^−10^, 0.007 and < 2 × 10^−16^, respectively. It should be noted that compared to the other programs CCMetagen and Metaphlan often reported species as 'unclassified' (although their genus classification was correct) accounting for their very low species recall. MetaPhlAn2 was unable to correctly classify any of the expected eukaryotes and therefore recall and precision could not be calculated.

**Table 3:** Mean (standard deviation) of recall of each classifier for bacteria, viruses and eukaryotes using the unordered approach

Classifier	Bacteria	Virus	Eukaryotes
BURST	0.86 (0.017)	0.664 (0.045)	0.514 (0.04)
Kraken2	0.852 (0.015)	0.697 (0.057)	0.748 (0.049)
Centrifuge	0.571 (0.02)	0.626 (0.045)	0.69 (0.026)
CCMetagen	0.347 (0.022)	0.566 (0.043)	0.07 (0.022)
MetaPhlAn2	0.396 (0.039)	0.237 (0.042)	–

**Table 4:** Mean (standard deviation) of precision of each classifier for bacteria, viruses and eukaryotes using the unordered approach

Classifier	Bacteria	Virus	Eukaryotes
BURST	0.684 (0.029)	0.623 (0.038)	0.716 (0.05)
Kraken2	0.586 (0.033)	0.698 (0.036)	0.589 (0.035)
Centrifuge	0.467 (0.032)	0.673 (0.029)	0.452 (0.019)
CCMetagen	0.692 (0.038)	0.638 (0.05)	0.328 (0.098)
MetaPhlAn2	0.372 (0.04)	0.536 (0.076)	–

For bacteria, the highest mean values for precision (with overestimation of species number being penalised) were achieved similarly with CCMetagen (0.692) and BURST (0.684) followed by Kraken2 (0.586), Centrifuge (0.467) and MetaPhlAn2 (0.369), with *P*-values = 0.64, 1.24 × 10^−6^, 1.7 × 10^−7^ and 4.3 × 10^−12^, respectively. For virus precision, Kraken2 performed best (0.698), followed by Centrifuge (0.673), CCMetagen (0.638), BURST (0.623) and MetaPhlAn2 (0.536), with corresponding *P*-values = 0.09, 0.08, 0.45 and 0.005, respectively. For eukaryote precision, BURST (0.716) performed best followed by Kraken2 (0.589), Centrifuge (0.452) and CCMetagen (0.328), with corresponding *P*-values = 4 × 10^−6^, 2 × 10^−9^ and 0.001, respectively.

### Effect of taxonomic order of analysis

**Fig. 3:**
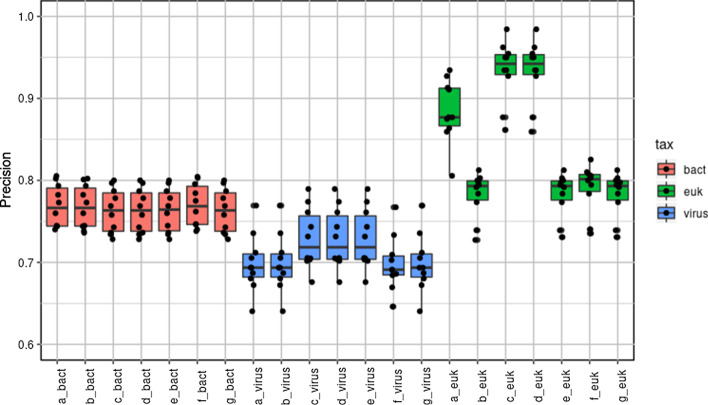
Precision determined with BURST classifier for each taxonomic group, using seven different orders of analysis: a = viruses > bacteria > eukaryotes; b = viruses > eukaryotes > bacteria; c = bacteria > eukaryotes > viruses; d = bacteria > viruses > eukaryotes; e = eukaryotes > bacteria > viruses; f = eukaryotes > viruses > bacteria; g = unordered

BURST performed consistently well compared to other programs in unordered analysis and therefore was chosen to examine the effect of ordering the analysis in six different ways compared to unordered analysis. Figure 3 shows box plots of precision for each ordered method, a–f, and unordered method, g. For bacteria, changing the order of analysis had no significant effect (*P* = 0.992). For viruses, orders c, d and e significantly improved precision (c, d, e > a, b, f, g; *P* = 0.001). For eukaryotes, orders a, c, and d significantly improved precision (a, c, d > b, e, f, g; *P* < 2 × 10^−16^). All orders of analysis which improved precision placed bacterial classification and removal of bacterial classified reads before the classification of viruses (c, d and e) or eukaryotes (a, c and d).

**Fig. 4:**
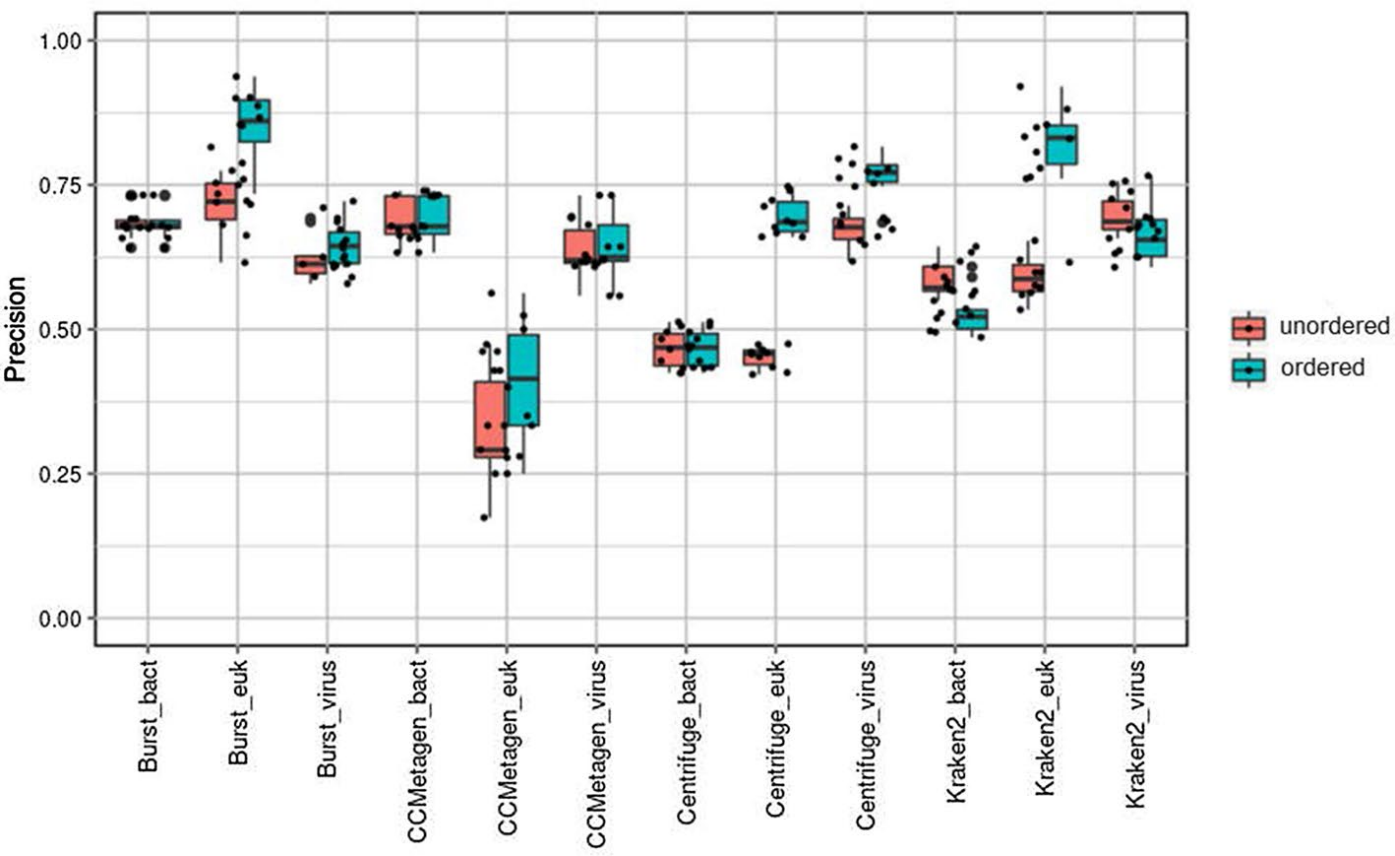
Precision determined by each classifier program after ordered analysis ‘d’ (bacteria > viruses > eukaryotes) and unordered analysis

Figure 4 shows a comparison of ordering analysis by method ‘d’ (bacteria > viruses > eukaryotes) for each classifier and each taxonomic group. Order ‘d’ had no effect on bacterial classification (*P* = 1.000). For virus classification, order ‘d’ significantly improved precision for Centrifuge (*P* = 4 × 10^−6^) but not for BURST, CCMetagen or Kraken2 (*P* = 0.145, 0.888, 0.143, respectively). For eukaryote classification, order 'd' significantly improved precision for BURST, Centrifuge and Kraken2 (*P* = 3 × 10^−5^, 7 × 10^−14^ and 5 × 10^−10^, respectively) but not for CCMetagen (*P* = 0.072). Precision means (standard deviation) for each classifier with order ‘d’ analysis are summarised in Table 5.

**Table 5:** Precision mean (standard deviation) determined with each classifier program after ordered analysis ‘d’ (bacteria > viruses > eukaryotes)

Classifier	Bacteria	Viruses	Eukaryotes
BURST	0.684 (0.029)	0.65 (0.041)	0.854 (0.06)
Kraken2	0.586 (0.033)	0.667 (0.053)	0.828 (0.052)
Centrifuge	0.467 (0.032)	0.767 (0.035)	0.696 (0.033)
CCmetagen	0.692 (0.038)	0.642 (0.051)	0.413 (0.1)

### Effect of sequencing depth

**Fig. 5:**
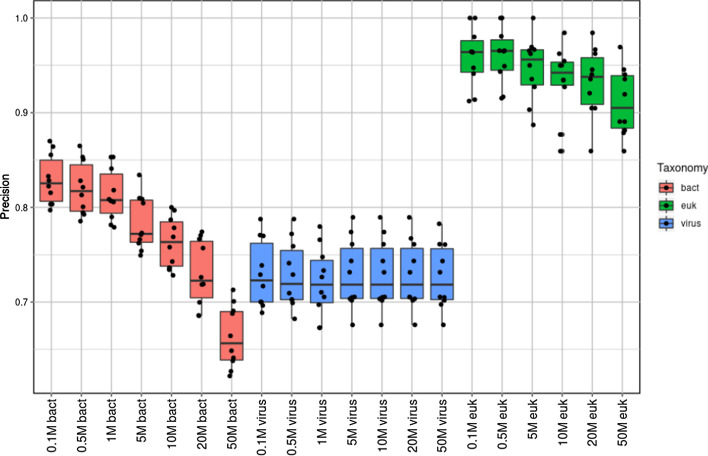
Effect of sequencing depth on precision determined by BURST classifier for each taxonomic group, ordered using method ‘d’ (bacteria > viruses > eukaryotes)

**Fig. 6:**
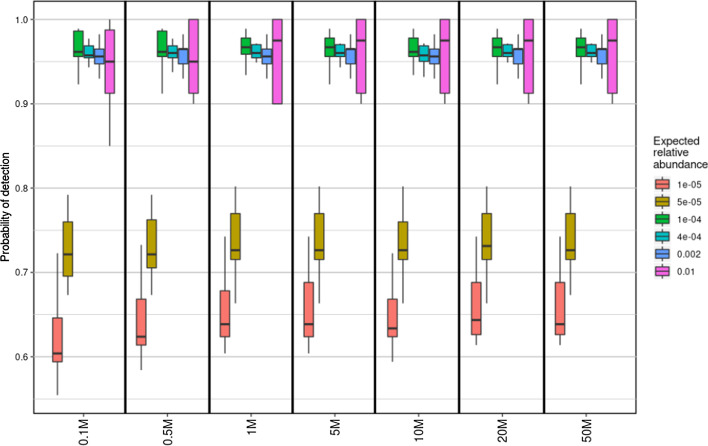
Effect of increasing sequencing depth (grouped on x-axis) on the probability of detecting and classifying species within six expected relative abundance bins (key denotes lower limit of bin)

The effect of sequencing depth (number of simulated reads classified per replicate) is shown in Fig. 5 for the BURST program, using ordered analysis method ‘d’. Bacterial precision decreased significantly with increasing depth (R^2^ = 0.745, *P* < 2 × 10^−16^), as did eukaryotic precision to a lesser extent (R^2^ = 0.187, *P* = 5.5 × 10^−4^). Increasing sequencing depth had no effect on virus classification precision (R^2^ = 1.3 × 10^−5^, *P* = 0.976). Sequencing depth had no effect on the probability of correctly classifying species (any species within the expected abundance bins shown) at an expected abundance of 1 × 10^−5^ or above (R^2^ = 0.02, *P* = 0.227) (Fig. 6). Note that error bars for ≥ 0.01 relative abundance are large due to few species being within this abundance bin (approximately five per simulation).

## Discussion

Our findings lead us to conclude overall that existing programs designed for the taxonomic classification of metagenomic data can classify non-bacterial sequences in fecal DNA, although precision varies considerably between programs. Moreover, when classifying eukaryotes, without considering the order of analysis, some programs performed unsatisfactorily. Ordering the analysis, so that bacterial reads were classified first and removed from subsequent classification of viruses and eukaryotes, significantly increased precision for eukaryotes. All the classifier programs evaluated suffered from over-classification, that is, they identified more species than expected in the sample, and this was only accentuated when sequencing depth was increased.

The speed of classifier programs is an important consideration when analysing large numbers of samples. Even small differences in speed will be amplified and speed may therefore be a deciding factor in choice of program. We found that Centrifuge was the fastest classifier, although at the cost of some precision as discussed below. Despite using a smaller, marker gene-based database, MetaPhlAn2 was the third fastest classifier. In addition to speed, database size and the RAM required is also an important factor in choice of classifier. The eukaryote databases used were very large and the different indexing and compression methods of each program resulted in sizes ranging from 115 to 218 GB, well beyond the RAM capacity of most desktop computers. Eukaryote classification is therefore currently not possible with whole genome classifiers unless access to high-performance computing or cloud-computing is available, except where a marker-gene program such as MetaPhlAn2 is used or databases are constrained to particular genomes, e.g. mitochondria or chloroplasts.

The difference between the commonly used metrics for metagenomic benchmarking, recall and precision, is crucial when comparing programs. For example, a more conservative program such as CCMetagen gave comparatively poor recall (for bacteria) but with greatly improved precision because it didn’t over-classify species. Conversely, Kraken2, had a relatively high recall, but suffered in precision due to over-classification. We consider that precision is a preferable measure to recall, because in the absence of any known estimate of the number of expected species in a sample, over-classification could yield misleading results. The problem of over-classification is usually addressed by bioinformaticians by excluding or filtering taxa that are low abundance, e.g. at an arbitrary value of 100. While this improves precision, it is not usually possible to define the correct level of filtering a priori and, as shown, this may be influenced by sequencing depth.

When analyses were unordered, the BURST program gave the best performance overall across bacteria, viruses and eukaryotes. Unlike the other classifiers, BURST uses full-length alignment of reads for classification, and not k-mers. Although this approach is thought to be too slow for classification of large read numbers BURST achieved comparable speeds to k-mer aligners. Kraken2 performed equally well to BURST in terms of recall and significantly better than BURST for eukaryote classification, but in terms of precision suffered from over-classification, reducing its relative performance. This effect could be mitigated by filtering lower abundance species from Kraken2, as outlined below. The absolute levels of recall and precision observed serve to highlight that even in ideal conditions, the classifiers tested will give approximately 27% misclassification at the species level (based on BURST ordered analysis average precision over all taxonomic groups). Accordingly, the identity of assigned species should be manually checked if they are considered to be important, for example with respect to differential abundance among samples. However, the species classification obtained (tens to hundreds of species) greatly reduces the number of such checks required compared to the numbers of original sequence reads, making manual checks with, e.g. BLAST, against complete online nucleotide databases feasible. The comparison of classification programs in this study used an 'ideal case' of simulated reads taken from the same sets of genomes as used to construct their databases. In real studies of naturally occurring metagenomes, where species will have sequence differences to those in the databases and will contain unknown species, we would therefore expect that recall and precision would be worse than shown here. However, at higher taxonomic levels this would be less of a problem. In this study we elected to compare only species to keep comparisons between programs and approaches simpler.

Eukaryote genomes are known to contain viral and bacterial sequences, due to physical contamination during sequencing and through actual genomic integrations. Similarly, bacterial genomes contain many phage and other virus-like sequences. It is therefore possible that these could cause erroneous read classifications, particularly in the case of eukaryotes in fecal samples, expected to be in low abundance compared to bacteria. For this reason, we examined the effect of changing the order of analysis to sequentially remove potentially ‘false’ reads. As expected, this did not affect bacterial classification, but the effect on viral and eukaryotic classification was significant. Virus precision was consistently increased when bacterial reads were classified first and removed prior to viral classification. Similarly, eukaryotic classification was increased to an even greater extent when bacterial reads were classified first and removed. We therefore recommend that future virus and eukaryote classification methods adopt a similar approach. The effect of ordering the analysis with bacterial classification first, followed by viruses and then eukaryotes, was greatest with the Kraken2 program, making it the most precise for eukaryote classification.

Sequencing depth had an unexpected effect on classification. With the BURST program, which performed consistently well in benchmarking, increasing the sequencing depth, from 1 × 10^5^ to 5 × 10^7^ reads led to a significant decrease in the precision of bacterial and, to a lesser extent, eukaryotic classification. Thus, while theoretically increasing the probability of detecting rarer species, increasing sequencing depth leads to an increase in classification of reads into species not present in the sample. Furthermore, increasing sequencing depth did not significantly increase the probability of detecting and classifying rarer species. The probability fell below 0.95 at an approximate abundance of 1 × 10^−4^, regardless of sequencing depth. We therefore conclude that increasing sequencing depth beyond approximately one million reads per sample has little advantage and may result in increased misclassification and over-classification at the species level.

## Conclusions

At the species level, the choice of metagenome classifier program had a marked effect on the precision of species assignment in different taxonomic groups. We found that overall, BURST, a program that uses full-length read alignment, was most precise across bacteria, viruses and eukaryotes. Kraken2 (a k-mer aligner) performed similarly well, and was programmatically more straightforward to use than BURST, but tended to over-classify reads more than BURST. This tendency could be remedied by filtering of low abundance species. The order in which taxonomic classification was performed markedly improved precision. There was a significant improvement when bacterial classified reads were filtered and removed from subsequent virus and eukaryote classification. Increased sequencing depth was found to decrease classification precision because it caused the programs studied to over-classify species. Furthermore, increased sequencing depth did not give a significant improvement in the probability of detection of a species below 1 × 10^−4^ relative species abundance, and therefore we conclude that sequencing depth for fecal metagenomes beyond five million reads per sample is not advisable for applications where the goal is to obtain microbiome, virome, or eukaryome population profiles.

## Methods

### Classifier programs

Five metagenome taxonomy classifier programs were selected for this study: BURST (v1.0) [11]; Kraken2 (v2.0.7) [12]; Centrifuge (v1.0.4) [13]; CCMetagen (v1.2.3) [10]; and MetaPhlAn2 (v2.9) [14]. A sixth classification pipeline using UBLAST [15] was also tested but proved too slow for practical use and was not examined further. While we refer to the programs collectively as 'classifiers' they may encapsulate several methods in order to achieve taxonomic classification of sample DNA reads, e.g. sequence alignment, database compression and indexing, and taxon filtering and selection such as lowest common ancestor (LCA) algorithms. BURST is a recently developed classifier which, unlike others available, uses a full-length alignment method (similar to BLAST) while achieving speed comparable to k-mer aligners. Kraken2, a k-mer based aligner, is the second generation of the very widely cited Kraken software for metagenomic classification. Centrifuge, another widely cited classifier, is meant to require less memory for its databases than Kraken2. CCMetagen is a recent classifier which uses a rapid k-mer mapping tool, KMA [16] to align and assign confidence to classified reads. CCMetagen's authors report that it is particularly suited to classification of eukaryotes. MetaPhlAn2 is very widely used and regarded as the benchmark for bacterial metagenome classification. Unlike the other classifiers in this study, MetaPhlAn2 uses a curated database of marker genes, rather than user-supplied sequences or genomes. Although MetaPhlAn2 is focussed on bacterial classification it also contains viruses and eukaryotes in its database. All programs were used with default settings where possible (see Additional file 1). The output of BURST consisted of all read alignments above the specified threshold but did not include a best-classification algorithm or read counts per taxon. A Python-based LCA and counting script was therefore written for this purpose (included in Additional file 1). Separate databases were built for each program for each taxonomic group: bacteria (and archaea), viruses, and eukaryotes. For the purposes of comparison, we examined classification performance to the species level.

### Databases and metagenome simulations

With the exception of MetaPhlAn2, all classifiers were provided with an identical set of genomes for building bacterial, viral, and eukaryotic databases, using the default instructions in their respective manuals. Genomes were downloaded from the NCBI RefSeq database (November 2019) using only representative genomes. These comprised 3,456 bacteria (and archaea), 3,939 viruses and 379 eukaryotes, with total lengths of 13.9, 0.104, and 32 Gbp, respectively. In order to simulate metagenomic sequence reads containing realistic numbers of representatives from bacteria, viruses and eukaryotes, existing simulation software e.g. MetaSim [17], could not be used because it is designed only for bacterial genomes. Large eukaryotic genomes cannot be accommodated because the software requires the frequency of whole genomes in simulations to be specified and total observed read abundances of eukaryotes would constitute only fractions of genomes. We therefore wrote new Python- based scripts (see Additional file 1) which simulated bacterial, viral and eukaryote abundance on a *per read* basis from distributions observed in our experimental observations of the abundances of such reads in fecal metagenomes in our laboratory [18]. Logarithmic and exponential decay models were manually evaluated for best-fit to the observed metagenome species abundances. The best fitting simple model was found to be an exponential decay relationship of abundance to species rank: species abundance = (1.5 × rank) ^−2^ (R^2^ = 0.948). See supplementary data file "Abundance_model_fit.xlsx". It should be noted that we do not expect this distribution to apply generally to metagenomes, but it showed a good fit to our observed data. The purpose of this fitted distribution was only to generate realistic simulations of metagenome species abundance for the purposes of this study. The simulated reads incorporated a recently described error rate for Illumina sequencing [19]. Ten replicate simulations were performed for each program/condition studied. With the exception of the sequencing depth analysis described below, all simulations were performed with a target number of 10 million single-ended reads from 300 bacteria, 100 viruses and 100 eukaryotes. In order to simulate the expected distribution curve for each taxon, exact numbers for total reads and numbers of taxa could not be used. Numbers achieved were ± 10% of the target. For statistical calculations, the actual simulated numbers were used, not the targets. Single ended reads were used because Burst could not use paired-read information. We also found in initial studies that paired-read classifications were not more accurate when using Kraken2 alone, but increased processing times (results not shown).

### Classifier program runs

All scripts and pipelines are provided in the Additional file 1. Analyses were carried out on the Walter and Eliza Hall Institute High Performance Computing Cluster, using 24 × Intel(R) Xeon(R) Gold 6130 CPUs @ 2.10 GHz. The same compute resource of 24 CPUs, and available RAM of 256 GB, was used for all programs / conditions. For each program, seven different orders of taxonomic group analysis were tested: a = viruses > bacteria > eukaryotes; b = viruses > eukaryotes > bacteria; c = bacteria > eukaryotes > viruses; d = bacteria > viruses > eukaryotes; e = eukaryotes > bacteria > viruses; f = eukaryotes > viruses > bacteria; g = unordered (all reads classified against all databases). It was expected that ordering analyses in this way could help to reduce misclassification of viruses and eukaryotes when reads arise from exogenous sources (integrated virus or bacterial sequence) or from homologues within eukaryotes. This approach was used because preliminary analyses showed that mis-classified eukaryotic reads could originate from ‘bacterial’ sequences that resulted from either contamination or integration events known to be present in the RefSeq genomes [20]. At the second and third step of ordered analysis, reads classified by the previous steps were excluded; however, this was not possible with MetaPhlAn2 which therefore was only used in the unordered mode. It should be noted that using the above methodology, the comparisons drawn below are biased against MetaPhlAn2 because its marker gene database was not specifically designed for, or drawn from the RefSeq genomes used in this study (as was the case for all other classifiers studied). MetaPhlAn2 was included because it is very widely used, e.g. in the Human Microbiome Project [21]. Additionally, MetaPhlAn3, which was not available at the time of these analyses, may provide improvements.

The effect of sequencing depth (total number of simulated reads) was examined with BURST, using the analysis order bacteria > viruses > eukaryotes, with 50, 20, 10, 5, 1, 0.5 and 0.1 million reads. In this analysis, we also examined the probability of correct species classification (any taxonomic group) at six relative abundance bin ranges: > 0.01, 0.01–0.002, 0.02–4 × 10^−4^, 4 × 10^−4^–1 × 10^−4^, 1 × 10^−4^–5 × 10^−5^ and < 1 × 10^−5^. BURST was chosen because in preliminary work its performance was consistently better than other classifiers. We expect trends observed using BURST to be similar for other classifiers.

The performance of classifier runs was assessed using two previously described and widely used statistics: recall and precision [22].^1^ Recall is defined as the number of correctly identified taxa divided by the total expected number. Precision is defined as the number of correctly identified taxa divided by the total number classified. Recall can therefore be biased to methods that classify more species, and false positives (over-classification) because it does not take the total number classified into account. Precision will penalise methods which over-classify but in real use situations rare taxa are usually filtered from results, for which precision does not account. However, in this study we were interested specifically in differences between programs in the rarer components of metagenomes, while minimising over-classification. Therefore, for our purposes, precision may be considered the more useful statistic. We only considered classification to the species level in this study in order to simplify analyses, and with respect to the classification of eukaryotes, e.g. foods and parasites, we consider species level classification the most useful classifier result and we have therefore only considered the species level in this work. It may be the case that the relative differences observed between programs and conditions could vary when higher taxonomic levels are used. Differences and trends in results were assessed with ANOVA and linear models using the R package (v3.6.1).

## Supplementary Information

**Additional file 1**. File containing all scripts and information for benchmarking simulations. This information is also available at the link: https://github.com/theo-allnutt-bioinformatics/Allnutt_etal_2020_expanding_the_taxonomic.

## Data Availability

All data generated or analysed during this study are included in this published article and its supplementary information files, which are available in the repository: https://github.com/theo-allnutt-bioinformatics/Allnutt_etal_2020_expanding_the_taxonomic

## Abbreviations

ANOVA: Analysis of variance
Bp: Base pairs
CPU: Central processing unit
DNA: Deoxyribonucleic acid
GB: Gigabytes
Gbp: Giga-basepairs
GHz: Gigahertz
LCA: Lowest common ancestor
NCBI: National Center for Biotechnology Information
RAM: Random access memory
RNA: Ribonucleic acid
rRNA ITS: Ribosomal ribonucleic acid intergenic transcribed spacer

## Footnotes

1 In this paper we conform to the previous benchmarking use of the term 'precision' for the statistic. However, we recognise that the term is a measure of accuracy.
